# The FEV1/DLCO Ratio as an Effective Predictor of Severity and Survival in COPD-Associated Pulmonary Hypertension: A Retrospective Analysis

**DOI:** 10.3390/jcm14051606

**Published:** 2025-02-27

**Authors:** Ria Patel, Jay Pescatore, Shameek Gayen

**Affiliations:** 1 Department of Internal Medicine, Temple University Hospital, Philadelphia, PA 19140, USA; ria.patel@tuhs.temple.edu; 2 Department of Thoracic Medicine and Surgery, Lewis Katz School of Medicine, Temple University Hospital, Philadelphia, PA 19140, USA; jay.pescatore@tuhs.temple.edu

**Keywords:** pulmonary hypertension, chronic obstructive pulmonary disease, FEV1, DLCO

## Abstract

**Background/Objectives**: Pulmonary hypertension (PH) is associated with increased morbidity and mortality in chronic obstructive pulmonary disease (COPD). The ratio of the functional vital capacity (FVC) to diffusing capacity of the lung for carbon monoxide (DLCO) has demonstrated predictive and prognostic efficacy in PH due to lung disease, including COPD. However, forced expiratory volume in 1 s (FEV1) is used to grade COPD severity. We aimed to determine whether FEV1/DLCO predicts PH severity in COPD-PH. **Methods**: This is a retrospective analysis of patients with COPD-PH diagnosed via right heart catheterization and pulmonary function testing. Linear regression assessed the correlation of FEV1/DLCO with RHC parameters. Receiver operating characteristic (ROC) analysis was performed to assess the predictive effectiveness of FEV1/DLCO for severe PH. **Results**: Among 212 patients with COPD-PH, the FEV1/DLCO ratio positively correlated with mean pulmonary artery pressure (mPAP; r = 0.6, *p* < 0.001) and pulmonary vascular resistance (PVR; r = 0.56, *p* < 0.001). In ROC analysis, FEV1/DLCO was effective at predicting severe PH (AUC 0.60, *p* = 0.02). Those with a FEV1/DLCO ratio > 1.66 had a decreased rate of transplant-free survival as compared to those with a lower ratio (40.8% vs. 59.2%, *p* = 0.01). **Conclusions**: Among patients with COPD-PH, FEV1/DLCO correlates well with mPAP and PVR. The FEV1/DLCO ratio may effectively predict severe PH and may predict transplant-free survival in COPD-PH.

## 1. Introduction

Pulmonary hypertension (PH) is a prevalent comorbidity associated with chronic obstructive pulmonary disease (COPD). Up to 90% of patients with Global Initiative for Chronic Obstructive Lung Disease (GOLD) Stage 4 have a mean pulmonary artery pressure (mPAP) greater than 20 mmHg [1]. The presence of PH in COPD is associated with increased morbidity, mortality, and healthcare utilization [2,3,4].

While PH is diagnosed via right heart catheterization (RHC), as defined by mean pulmonary artery pressure [mPAP] > 20 mmHg, several non-invasive measures have been validated in predicting and prognosticating PH. Although RHC remains the gold standard for diagnosis, it is not always necessary for guiding treatment or predicting prognosis, especially in patients with PH associated with lung disease, typically classified as World Health Orgaization (WHO) Group 3 PH. In this subgroup of PH patients, non-invasive testing has become increasingly important. One such measure, specifically used in chronic lung disease and PH, is the forced vital capacity (FVC)/diffusing capacity of the lung for carbon monoxide (DLCO) ratio [5]. The decreased FVC in COPD patients can be attributed to emphysematous destruction and distal small airways disease. The destruction of the capillary bed surface area contributes to decreased DLCO in these patients as well [6]. The FVC/DLCO ratio has shown utility in diagnosing patients with group 3 PH and obstructive lung disease. The FVC/DLCO ratio is higher in COPD patients with PH than those without PH and serves as a prognostic factor for 5-year all-cause mortality [5]. However, the severity of airflow obstruction in COPD is measured via forced expiratory volume in 1 s (FEV1), not FVC. It is incorporated into essential indices in COPD, such as the BODE index for COPD mortality and the GOLD criteria for COPD severity [3,7].

The primary objective of this study was to determine whether FEV/DLCO correlates with PH and predicts PH severity in patients with COPD-PH. We hypothesize that FEV1/DLCO is effective in predicting PH severity and is superior to FVC/DLCO in doing so in patients with COPD-PH.

## 2. Materials and Methods

### 2.1. Study Design

A retrospective study was conducted with patients with COPD and PH at our institution between 2011 and 2023. Inclusion criteria included adult COPD patients greater than 18 years of age diagnosed with spirometry data at an accredited institution (FEV1/FVC < 0.70) and PH (mPAP > 20 mmHg). Severe PH was defined as PVR > 5 WU based on 2022 ESC/ERS guidelines for PH associated with chronic lung disease [8]. We collected data from RHC diagnostics of PH and pulmonary function testing performed closest to RHC diagnostics of PH. We excluded patients with PH associated with lung diseases aside from COPD and patients with PH due to other etiologies. In addition to spirometry and RHC data, we collected DLCO, 6 min walk distance (6MWD), and oxygen requirements. Our study received approval for the waiver of informed consent from the Western Institutional Review Board (IRB, Protocol # 31795). Procedures were performed in accordance with the ethical standards of the Western IRB and the Helsinki Declaration of 1975. The authors used the Strengthening the Reporting of Observational Studies in Epidemiology (STROBE) checklist in the preparation of this manuscript [9].

### 2.2. Statistical Analysis 

Continuous variables were expressed as means ± standard deviation (SD). Linear regression was performed to determine the correlation of the FEV1/DLCO ratio with mPAP, PVR, oxygen requirement, and 6MWD; Pearson’s correlation coefficient (R) was used to assess the correlations. Receiver operating characteristic (ROC) and area under the curve (AUC) analysis were used to determine the effectiveness of FEV1/DLCO and FVC/DLCO in predicting severe PH. The AUC was calculated with a 95% confidence interval (CI). The optimal cutoff point of FEV1/DLCO for predicting severe PH was determined using Youden’s index. Logistic regression was performed to determine the associated risk of developing severe PH with the determined FEV1/DLCO cutoff. Statistical significance was measured with a *p*-value < 0.05. Transplant-free survival was compared using a Chi-squared test, followed by logistic regression in which the risk of death or transplant associated with the determined FEV1/DLCO cutoff was assessed. Statistical analysis was performed with the use of IBM SPSS Statistics, Version 29. 

## 3. Results

**Figure 1:**
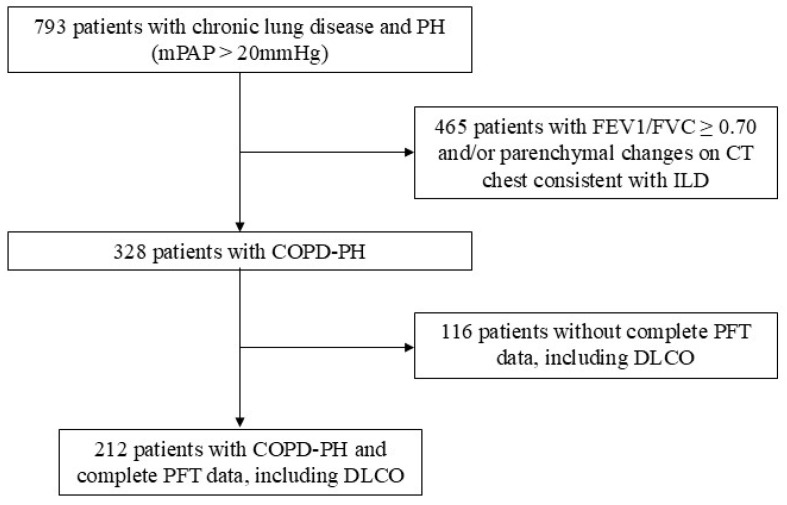
Patient cohort selection: we identified patients with COPD-PH and complete PFT data. COPD: chronic obstructive pulmonary disease; CT: computed tomography; DLCO: diffusing capacity of the lung for carbon monoxide; FEV1: forced expiratory volume in 1 s; FVC: forced vital capacity; ILD: interstitial lung disease; mPAP: mean pulmonary artery pressure; PFT: pulmonary function testing; PH: pulmonary hypertension.

We identified 212 patients with COPD and PH (Figure 1); demographic and clinical characteristics can be seen in Table 1. Notably, the average mPAP was over 30 mmHg, with an average PVR of 4.2 WU. Among the cohort, 51 patients had severe PH, or PVR > 5 WU. The average FEV1 was predicted to be 52.7%. The average DLCO was predicted to be 26.4%. The average 6MWD was 251.5 m, and the average oxygen requirement was 5.7 L/min (Table 1).

**Figure 2:**
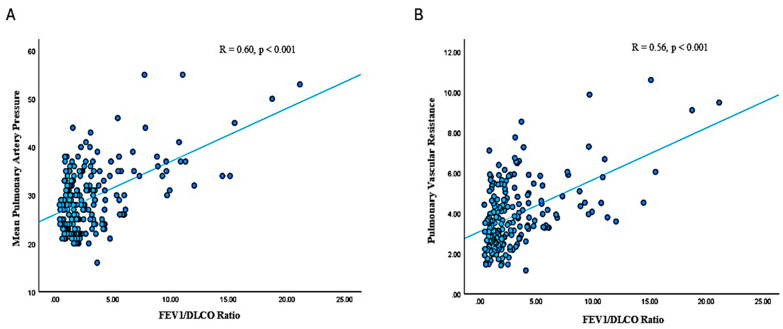
Association of FEV1/DLCO with RHC parameters. (**A**) Association between FEV1/DLCO and mPAP. (**B**) Association between FEV1/DLCO and PVR. DLCO: diffusing capacity of the lung for carbon monoxide; FEV1: forced expiratory volume in 1 s; mPAP: mean pulmonary artery pressure; PVR: pulmonary vascular resistance.

**Figure 3:**
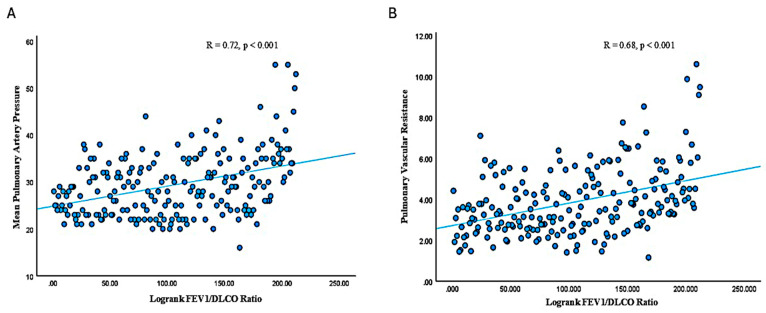
Association of logrank FEV1/DLCO with RHC parameters. (**A**) Association between logrank FEV1/DLCO and mPAP. (**B**) Association between logrank FEV1/DLCO and PVR. DLCO: diffusing capacity of the lung for carbon monoxide; FEV1: forced expiratory volume in 1 s; mPAP: mean pulmonary artery pressure; PVR: pulmonary vascular resistance.

Linear regression analysis was used to correlate the FEV1/DLCO ratio with various hemodynamic and clinical parameters (Figure 2). The FEV1/DLCO had a significant positive correlation with mPAP (R = 0.60, *p* < 0.001), PVR (R = 0.56, *p* < 0.001), and the oxygen requirement (R = 0.59, *p* < 0.001). There was a trend towards negative correlation between FEV1/DLCO and 6MWD (R = −0.10, *p* = 0.17), but this was not significant. The logrank values of FEV1/DLCO (Figure 3) were also positively correlated with mPAP (R = 0.72, *p* < 0.001) and PVR (R = 0.68, *p* < 0.001).

**Figure 4:**
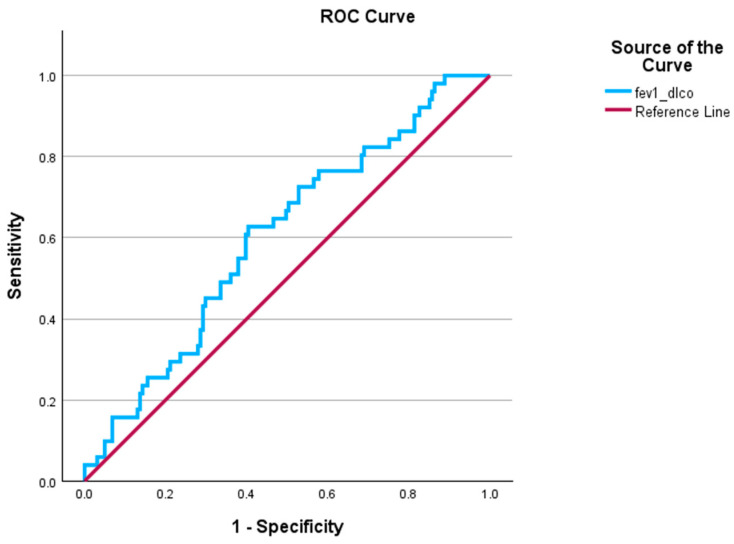
ROC for FEV1/DLCO ratio predicting severe PH. AUC = 0.60, *p* = 0.002; severe PH is defined as PVR > 5 WU. AUC: area under the curve; PH: pulmonary hypertension; ROC: receiver operator curve.

We then used ROC analysis to assess the ability of the FEV1/DLCO ratio to predict severe PH among patients with COPD-PH (Figure 4). The FEV1/DLCO ratio was a significant predictor of severe PH (AUC 0.60, *p* = 0.002).

**Figure 5:**
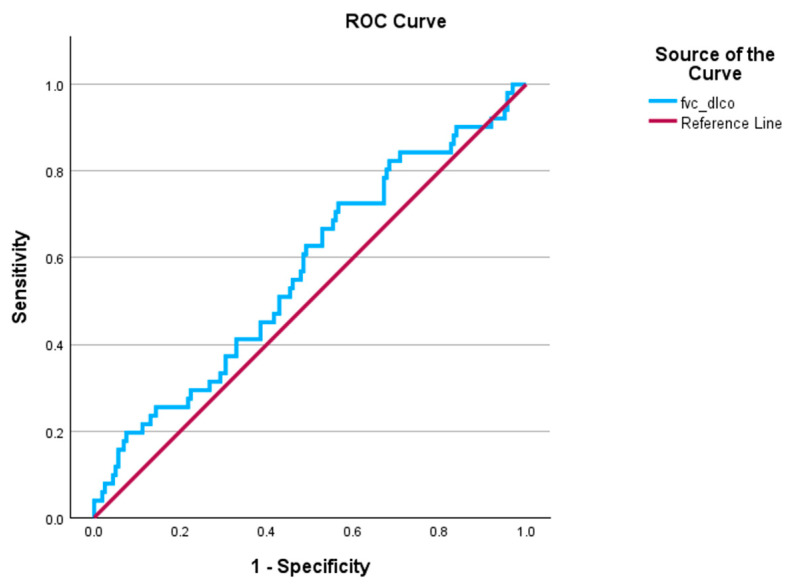
ROC for FVC/DLCO ratio predicting severe PH. AUC = 0.57, *p* = 0.14; severe PH is defined as PVR > 5 WU AUC: area under the curve; PH: pulmonary hypertension; ROC: receiver operator curve.

We then conducted ROC analysis to assess the ability of the FVC/DLCO ratio to predict severe PH among patients with COPD-PH (Figure 5); FVC/DLCO is not predictive of severe PH (AUC 0.57, *p* = 0.14).

The optimal cutoff of FEV1/DLCO was determined to be 1.66 via Youden’s index. Logistic regression analysis determined that an FEV1/DLCO ratio > 1.66 was significantly associated with severe PH (HR 2.36, 95% CI 1.19–4.70, *p* = 0.014), indicating that patients with COPD-PH and an FEV1/DLCO ratio > 1.66 have a greater than two-fold increased risk of developing severe PH. 

**Figure 6:**
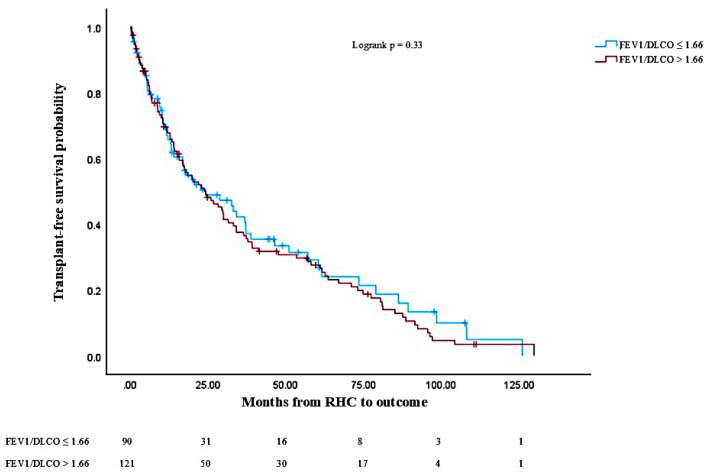
Kaplan–Meier transplant-free survival analysis. Kaplan–Meier survival analysis comparing transplant-free survival probability between COPD-PH patients with FEV1/DLCO > 1.66 and FEV1/DLCO ≤ 1.66. Number of patients at risk displayed at bottom of graph, and changes in graph due to either death or lung transplantation.

Transplant-free survival was compared via a Chi-square test. Patients with an FEV1/DLCO ratio > 1.66 had significantly worse transplant-free survival compared to those with a ratio ≤ 1.66 (40.8% vs. 59.2%, *p* = 0.01). Median follow-up was 24.2 months. Kaplan–Meier (Figure 6) survival analysis showed a trend towards worse transplant-free survival probability (logrank *p* = 0.33) in patients with FEV1/DLCO > 1.66. Cox regression showed a trend towards an increased risk of death or transplant among patients with FEV1/DLCO > 1.66 (HR 1.42, 95% CI 0.85–1.78, *p* = 0.33).

## 4. Discussion

We found that FEV1/DLCO positively correlates with mPAP and PVR among patients with COPD-PH. The FEV1/DLCO ratio also positively correlates with the oxygen requirement and has a trend towards negative correlation with 6MWD. Notably, FEV1/DLCO also effectively predicts severe PH, defined as PVR > 5 WU, in our cohort of patients with COPD-PH and is more effective in doing so than FVC/DLCO. An elevated FEV1/DLCO ratio is significantly associated with a greater than two-fold increased risk of severe PH. Transplant-free survival is lower in patients with an elevated FEV1/DLCO ratio.

Severe PH in chronic lung disease was previously defined as mean pulmonary artery pressure (mPAP) > 35 mmHg or mPAP ≥ 25 mmHg with cardiac index (CI) < 2.5 L/min/m [10]. However, the 2022 ESC/ERS guidelines for the diagnosis and management of PH have shifted to a PVR cutoff, with PVR > 5 WU adopted as the criteria for severe PH in chronic lung disease [8]. This shift was based on the literature showing that PVR > 5 WU is a better indicator of predicting a worse prognosis in COPD patients [11]. The identification of non-invasive measures that can effectively predict the development of severe PH is important, as early identification can prompt sooner intervention and potentially improve outcomes in patients with COPD-PH.

In COPD-PH, there are a few identified factors that predict outcomes: male sex, a low 6 min walking distance, a history of coronary artery disease, and elevated PVR [12,13]. Those who have severe PH as opposed to moderate PH have worse outcomes those with COPD [13,14]. It has also been shown that any elevation in PA pressures can be linked to an increased risk of severe exacerbation of COPD in patients with moderate to severe disease [15,16,17]. While FVC/DLCO has demonstrated an ability to identify and prognosticate PH in patients with COPD-PH, it does not fully capture the severity of airflow obstruction, as FEV1 is used to determine airflow obstruction severity in COPD [3,5]. Despite this, the utility of the FEV1/DLCO ratio in predicting severe PH has not been explored. In our study, FEV1/DLCO actually outperformed FVC/DLCO in predicting severe PH. This suggests that the FEV1/DLCO may best represent the relationship between airflow obstruction, gas exchange efficiency, and pulmonary vascular disease. This is owing to the pathophysiology of this COPD-PH cohort [4,11]. FEV1 has long been correlated directly with airflow obstruction, while DLCO reflects gas exchange efficiency as well as pulmonary vascular disease. These parameters, combined into a single ratio, can provide a comprehensive assessment of the relationship between both the underlying airflow obstruction and the vascular remodeling seen in COPD-PH patients, as well as allow the assessment of the complex relationship between many the pulmonary hemodynamics that interplay in the lung physiology seen in COPD. The significant correlation of this ratio with mPAP, PVR, and the oxygen requirement strengthens its capability as a prognostic and clinical tool.

In addition to predicting severe PH, this study also found that transplant-free survival was worse among COPD-PH patients with an elevated FEV1/DLCO ratio. This association highlights the bifunctional value of the FEV1/DLCO ratio: it can serve as a marker for severe PH as well as a potential mortality and transplant indicator.

This study has several limitations. As it is a retrospective study, we cannot control for selection bias and other factors. This includes possible confounding factors such as population demographics, environmental factors, and comorbid conditions. Additionally, the study’s size may limit the ability to generalize these results to other populations. Furthermore, this study was conducted at one institution, which may limit its generalizability. More prospective multicenter studies will be useful to validate the FEV1/DLCO ratio as a predictive marker across more patient cohorts. It would be interesting to assess the association between this ratio and other group 3 PH patients without COPD, as well as those without PH. It would be beneficial to explore its prognostic value in targeted endpoints such as quality of life, pulmonary vasodilator treatment response, and mortality since this study focused on limited clinical outcomes and pulmonary hemodynamics.

## 5. Conclusions

In conclusion, the FEV1/DLCO ratio may be an additional non-invasive tool that can aid in predicting severe PH and survival in patients with COPD-PH. It may provide a simpler, more accurate, and more accessible tool for the prediction of severe PH in patients with COPD-PH; when elevated, this can prompt further invasive diagnostic work-up. Although further studies are needed to validate its use clinically, the FEV1/DLCO ratio has the potential to further enhance risk classification and guide management for patients with COPD-PH.

## Figures and Tables

**Table 1:** Demographic and clinical characteristics of the cohort.

Age, years ± SD	64.8 ± 8.3
Biological Sex	
Male, n (%)	109 (51.4)
Female, n (%)	103 (48.6)
BMI, kg/m^2^ ± SD	28.9 ± 7.6
Race/Ethnicity	
White, n (%)	116 (54.7)
Black, n (%)	76 (35.9)
Hispanic, n (%)	14 (6.6)
Other, n (%)	6 (2.8)
RAP, mmHg ± SD	9.1 ± 4.6
Systolic PAP, mmHg ± SD	46.4 ± 16.7
mPAP, mmHg ± SD	30.8 ± 9.9
PCWP, mmHg ± SD	14.0 ± 5.5
CO, L/min ± SD	4.8 ± 1.4
CI, L/min/m^2^ SD	2.6 ± 0.7
PVR, WU ± SD	4.2 ± 3.2
Severe PH (PVR > 5 WU), n (%)	51 (24.1)
FEV1% predicted, % ± SD	52.7 ± 22.4
FVC% predicted, % ± SD	75.2 ± 19.9
DLCO% predicted, % ± SD	26.4 ± 14.5
6MWD, meters ± SD	251.5 ±101.7
Oxygen requirement, L/min ± SD	5.7 ± 4.8
Bronchodilator therapy	
LAMA, n (%)	24 (11.3)
LAMA-LABA, n (%)	30 (14.2)
LABA-ICS, n (%)	10 (4.7)
LAMA-LABA-ICS, n (%)	148 (69.8)

6MWD: 6 min walk distance; BMI: body mass index; CI: cardiac index; CO: cardiac output; DLCO: diffusing capacity of the lung for carbon monoxide; FEV1: forced expiratory volume in 1 s; FVC: functional vital capacity; ICS: inhaled corticosteroid; LABA: long-acting beta agonist; LAMA: long-acting muscarinic antagonist; mPAP: mean pulmonary artery pressure; PAP: pulmonary artery pressure; PCWP: pulmonary capillary wedge pressure; PVR: pulmonary vascular resistance; RAP: right atrial pressure.

## Data Availability

The data that support the findings of this study are available from the corresponding author upon reasonable request due to privacy reasons and to protect ongoing studies.

## Abbreviations

The following abbreviations are used in this manuscript:

MDPI	Multidisciplinary Digital Publishing Institute
DOAJ	Directory of open access journals
TLA	Three letter acronym
LD	Linear dichroism
6MWD	Six-minute-walk distance
AUC	Area under the curve
BMI	Body mass index
CI	Cardiac index
CO	Cardiac output
COPD	Chronic obstructive pulmonary disease
DLCO	Diffusing capacity of the lung for carbon monoxide
FEV1	Forced expiratory volume in 1 s
FVC	Forced vital capacity
IRB	Institutional review board
mPAP	Mean pulmonary artery pressure
PAP	Pulmonary artery pressure
PCWP	Pulmonary capillary wedge pressure
PH	Pulmonary hypertension
PVR	Pulmonary vascular resistance
RAP	Right atrial pressure
RHC	Right heart catheterization
ROC	Receiver operator curve
WHO	World Health Organization
